# Coronary Artery Disease Detection Using a Fuzzy-Boosting PSO Approach

**DOI:** 10.1155/2014/783734

**Published:** 2014-04-10

**Authors:** N. Ghadiri Hedeshi, M. Saniee Abadeh

**Affiliations:** Faculty of Electrical and Computer Engineering, Tarbiat Modares University, P.O. Box 14115-143, Tehran, Iran

## Abstract

In the past decades, medical data mining has become a popular data mining subject. Researchers have proposed several tools and various methodologies for developing effective medical expert systems. Diagnosing heart diseases is one of the important topics and many researchers have tried to develop intelligent medical expert systems to help the physicians. In this paper, we propose the use of PSO algorithm with a boosting approach to extract rules for recognizing the presence or absence of coronary artery disease in a patient. The weight of training examples that are classified properly by the new rules is reduced by a boosting mechanism. Therefore, in the next rule generation cycle, the focus is on those fuzzy rules that account for the currently misclassified or uncovered instances. We have used coronary artery disease data sets taken from University of California Irvine, (UCI), to evaluate our new classification approach. Results show that the proposed method can detect the coronary artery disease with an acceptable accuracy. Also, the discovered rules have significant interpretability as well.

## 1. Introduction

Accumulation of atherosclerotic plaques in coronary arteries leads to the coronary artery disease (CAD) which results in clogging of the coronary lumen, and, consequently, occlusion, and then leads to myocardial infarction (MI) or sudden cardiac death. The CAD is the leading cause of death in the United States. Understanding the pathophysiology of coronary artery disease, the prevention of its progression, the identification and efficient modification of cardiovascular risk factors, its diagnosis, and remedy in early and reversible phases is of great significance [1]. The “gold standard” method for the diagnosis of CAD, which is widely used, is coronary angiography (CA). However, CA is a costly and invasive procedure and needs technology and high-level technical experience; therefore, it cannot be used to screen large populations or close followup of treatment [2]. Hence, in the clinical setting, for the detection of CAD, other noninvasive methods are being used. The most important of those include exercise electrocardiogram (ECG) [3] testing, single photon emission computed tomography (SPECT or scintigraphy) [4], and stress echocardiography (ECHO), while multislice spiral computerized tomography (MSCT) or electron-beam computerized tomography (EBCT) and coronary magnetic resonance angiography (CMRA) are also being now used [2].

While many people with heart disease have symptoms such as angina, fatigue, and chest pain, many people have no symptoms until a heart attack happens. According to the American Heart Association (AHA), CAD is one of the most important killers of American men and women, reported as the cause for more than one of every five deaths in 2001 [5].

There are many risk factors related to CAD. Some factors such as family history, gender, and age cannot be controlled. However, other risk factors that are associated with lifestyle can often be controlled [6]. For example, physical inactivity, high cholesterol, high blood pressure, and smoking are all considered as risk factors for this disease that can be modified and even, in some cases, eliminated by modifying daily life routines and taking medication. Early changes in lifestyle can significantly prevent diabetes and obesity. The large number of factors that have to be analyzed for diagnosing CAD makes the physician's work even more difficult. In general, physicians make decisions by evaluating the existing test results of the patients. The earlier diagnoses made on other patients with the same condition are also considered by the physicians. These complicated procedures are not easy to perform when considering many factors that the physician has to evaluate. So, the decision about presence or absence of the disease depends on the physician's experience and skill to compare his patient with his previous ones. This procedure is a challenging task regarding the large number of factors that has to be considered. In this complex stage, the doctor may need an accurate tool that lists his earlier decisions about patient having the same (or close to same) factors [7].

During the past decades, the level of interest in the use of data mining and artificial intelligent tools in medical fields and the provision of healthcare has undergone a significant increase. Several sections of the researches in this area are related to developing the diagnostic tools that are used to help physicians in a diagnosis. As an advanced data mining technique, PSO has been applied to many tasks in medicine.

In PSO, particles are available to be adjusted by the learning process. In the research area of rule extraction and pattern recognition, this approach has been widely used. In this paper, we have applied PSO and fuzzy logic with a proposed boosting algorithm for the diagnosis of coronary artery disease.

The boosting mechanism adapts the distribution of training instances in a way that the previously misclassified or uncovered instances are further considered by the PSO algorithm.

The Cleveland, Hungarian, Switzerland, and VA Long Beach data sets, which are taken from Data Mining Repository of University of California, Irvine (UCI), have been used for testing this method [8].

The results show that this method can classify these data sets with acceptable accuracy or even better than the results achieved by previous works. This method is also superior to other methods in terms of interpretability.

The rest of this paper is organized as follows: a brief overview on related work, data set description, fuzzy rule-based classification system, particle swarm optimization, and ensemble based methods, is presented in the Background section. The proposed method involving a new boosting algorithm, fuzzy rule extraction with PSO, and, finally, En-PSO approach is discussed in Section 3. Experimental results are reported in Section 4, and the paper ends by some concluding remarks in Section 5.

## 2. Background

### 2.1. A Brief Overview on Related Works

Up to now, various researches have been done for the diagnosis of heart disease. These researches have used different methods for the detection of heart disease and have achieved relatively high accuracies, of 77% or higher on UCI machine learning repository data sets. Some examples are presented here.

Detrano et al. [9] applied a logistic-regression-derived discriminant function and achieved a correct classification accuracy of approximately 77%.

The accuracy achieved by John Gennari's CLASSIT conceptual clustering system was 78.9% on the Cleveland data set [10].

Gamboa et al. [11] proposed a fuzzy support vector clustering system to diagnose heart disease. In this method, a kernel induced metric was used to assign each piece of data. Experimental results were achieved with a well-known benchmark of heart disease.

Using SAS base software 9.1.3, an ensemble method with three neural networks was introduced by Das et al. [12]. The classification accuracy obtained by this method was 89.01% on Cleveland well-known data set.

Chau et al. [13] proposed the use of decision tree C4.5 algorithm, bagging with decision tree C4.5 algorithm, and bagging with Naïve Bayes algorithm to identify the heart disease of a patient and compare the effectiveness and correction rate among them. Results showed that bagging algorithms, especially bagging with Naïve Bayes, have the best performance among the tested methods.

In Rani's study [14], heart diseases data set is analyzed using neural network approach. To increase the efficiency of the classification process, the parallel approach is also adopted in the training phase. The experimental results showed that neural networks technique provides satisfactory results for the classification task.

A data set which has been called Z-Alizadeh Sani is introduced in [15]. This data set contains 303 patients and 54 features and introduces several effective features.

Acharya et al. [16] employed grayscale features from left ventricle echocardiographic images to classify patients with coronary artery disease.

*Data Set Description*. Coronary artery disease data sets are taken from Data Mining Repository of University of California, Irvine (UCI). The CAD data sets contain 920 instances collected from Cleveland, Hungarian, VA Long Beach, and Switzerland. Coronary angiography determines the result of CAD diagnosis. These data sets have 14 attributes of CAD data. These attributes are listed in Table 1. Following is a brief description of each of these data sets. 

*Cleveland Data*. Cleveland data set was collected by Robert Detrano, M.D. and Ph.D. degrees holder at V.A. Medical Centre. All the papers are related to the use of a subset of 14 of the 76 features that are presented in the processed Cleveland Heart Disease data set. The “end” field indicates the existence of coronary artery disease in the patients. This field includes an integer constant that can take any value from 0 to 4. Value 0 is for nonexistence and values 1, 2, 3, and 4 are for disease existence. In fact, these values indicate the number of blocked vessels. Six of the examples have missing values. Class distributions are 54% heart disease absent and 46% heart disease present.

#### 2.1.1. Hungarian Data

Andras Janosi, M.D. degree holder, collected this data set at the Hungarian Institute of Cardiology, Budapest. The format of this data set is the same as that of the Cleveland data. Class distributions are 37.5% heart disease present and 62.5% heart disease absent.

#### 2.1.2. Switzerland Data

This data set was collected at the University Hospital, Zurich, Switzerland, by William Steinbrunn, M.D. degree holder. Among four data sets related to CAD, the maximum number of missing value is related to Switzerland data set. It has 123 instances and class distributions in it are 6.5% heart disease absent and 93.5% with heart disease.

#### 2.1.3. VA Long Beach

VA Long Beach data set which has 200 instances was collected by Matthias Pfisterer, M.D. degree holder, at the University Hospital, Basel, Switzerland. This data set is in second rank in terms of the number of missing values. Class distributions in it are 25.5% heart disease absent and 74.5% heart disease present.

### 2.2. Fuzzy Rule-Based Classification System

Assume that we have a classification problem with *c* classes in the *n*-dimensional space with continuous attributes. Also, assume that *M* real vectors *x*_*p*_ = (*x*_*p*1_, *x*_*p*2_, …, *x*_*pn*_), *p* = 1,2,…, *m*, are given as training samples from the *c* classes (*c* ≪ *M*).

The pattern space is [0,1]^*n*^ and attribute values of each pattern are *x*_*pi*_ ∈ [0,1] for *p* = 1,2,…, *m* and *i* = 1,2,…, *n*. All the attribute values of each sample are in [0,1].

Fuzzy if-then rules, in this method, are expressed with the following form:

 rule *R*_*j*_: if *x*_1_ is *A*_*j*1_ and ⋯ and *x*_*n*_ is *A*_*jn*_, then class  *C*_*j*_ with CF = CF_*j*_.

Here, *R*_*j*_ is the label of the *j*th fuzzy if-then rule, *A*_*j*1_,…, *A*_*jn*_ are antecedent fuzzy sets in the range of [0,1], *C*_*j*_ is the result class (i.e., one of the given *c* classes), and CF_*j*_ is the degree of confidence of the fuzzy if-then rule *R*_*j*_.

In previous work [18], we used one uniform fuzzy partition for all attributes, while we know that these attributes may be of different kinds (e.g., continues, ordinal, and ratio). Therefore, it is more appropriate to apply several fuzzy partitions for various types of attributes. For example, in a binary attribute, only two fuzzy amounts are required, while, for a continuous attribute, more fuzzy amounts (like small, medium small, medium, medium large, and large) must be used.

**Figure 1:**
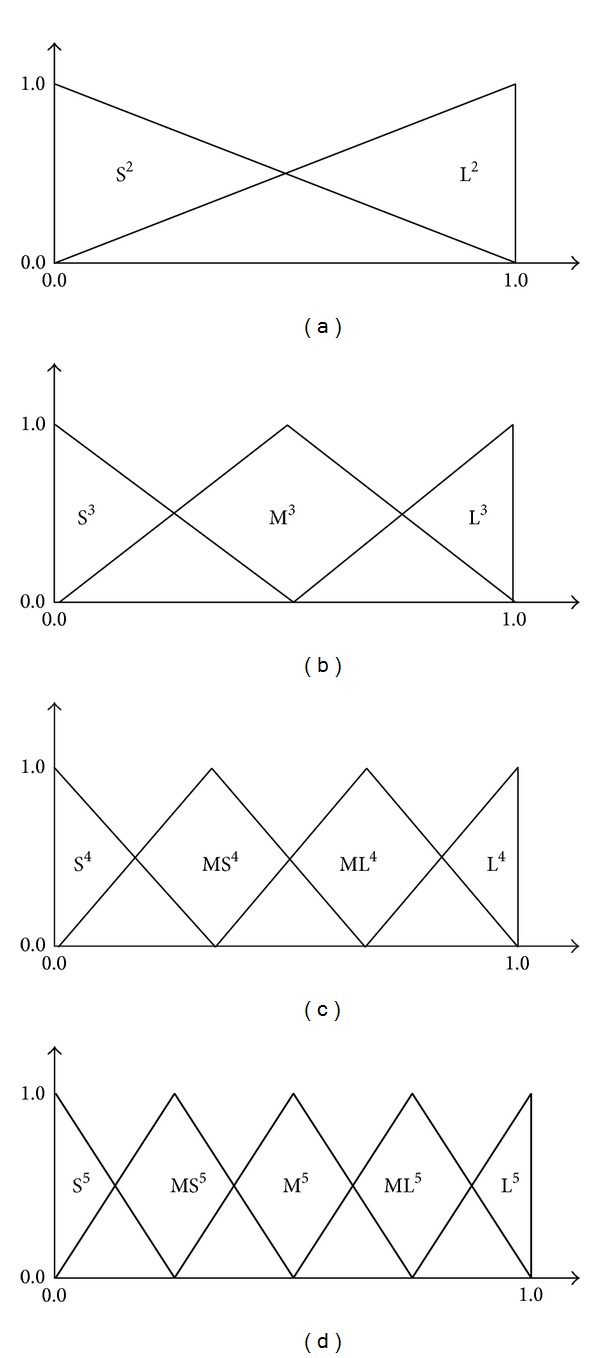
Four fuzzy partitions used in computer implementations. The superscript of each part shows the granularity of the fuzzy partition.

In computer implementations, we have used four fuzzy partitions evenly separated with symmetric triangular fuzzy sets in Figure 1.

To illustrate the high performance of our fuzzy classifier system, we use such simple specification in computer implementations.

However, we can use any adapted membership functions in our fuzzy classifier system for a specific pattern classification problem. When a rule is extracted with PSO, the following steps are applied to calculate the level of confidence of each fuzzy if-then rule.

**Step 1**

The compatibility of the fuzzy if-then rule *R*_*j*_ with each training instance *x*_*p*_ = (*x*_*p*1_, *x*_*p*2_, …, *x*_*pn*_) is obtained as follows:

(1) $$\begin{array}{c} \mu_{j}\left(x_{p}\right)=\mu_{j}\left(x_{p1}\right)\times \cdots \times \mu_{jn}\left(x_{pn}\right),\;p=1,2,\ldots ,m, \end{array}$$

where *μ*_*ji*_(*x*_*pi*_) is the membership function of *i*th attribute of *p*th instance and “*m*” indicates the total number of instances.

**Step 2**

For each of the classes, calculate the relative sum of the compatibility grades of the training instances with the fuzzy if-then rule *R*_*j*_:

(2) $$\begin{array}{c} \beta_{\text{Class}\;h}\left(R_{j}\right)=\underset{x_{p}\in \;\text{Class}\;h}{\sum }\frac{\mu_{j}\left(x_{p}\right)}{N_{\text{Class}\;h}},\;h=1,2,\ldots ,c, \end{array}$$

where *β*_Class *h*_(*R*_*j*_) denotes the sum of the compatibility grades of the training instances in Class *h* with the fuzzy if-then rule *R*_*j*_ and *N*_Class *h*_ is the number of training instances whose corresponding class is Class *h*.

**Step 3**

The certainty factor CF_*j*_ is calculated as follows:

(3) $$\begin{array}{c} {\text{CF}}_{j}=\frac{\left[\beta_{\text{Class}\;\overset{´}{h_{j}}}\left(R_{j}\right)-\left(\sum_{h\neq \overset{´}{h_{j}}}^{}\beta_{\text{Class}\;h}\left(R_{j}\right)/\left(c-1\right)\right)\right]}{\sum_{h=1}^{c}\beta_{\;\text{Class}\;h}\left(R_{j}\right)}. \end{array}$$

Now, we can determine the certainty factor for all combinations of antecedent fuzzy sets.

**Figure 2:**
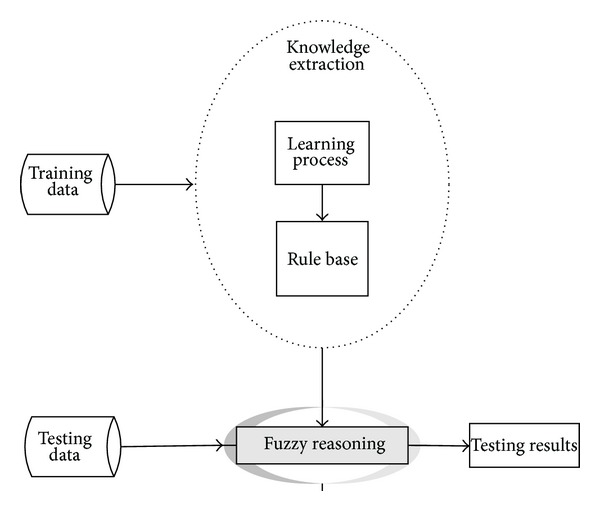
The basic model of rule-based fuzzy classifiers [17].

A fuzzy classification system (FCS) includes two basic steps [17]: (1) knowledge extraction which contains a set of fuzzy rules as extracted knowledge and (2) inference engine which classifies the input samples according to fuzzy rule set and reasoning method.  Figure 2 presents the basic model of rule-based fuzzy classification systems.

The goal of our fuzzy classification system (FCS) is to produce combinations of antecedent fuzzy sets for extracting a rule set S with more power to classify. With a rule set S, an input instance *x*_*p*_ = (*x*_*p*1_, *x*_*p*2_, …, *x*_*pn*_) is classified by a single winner rule *R*_*j*_ in S, which is determined by the following equation:

(4) $$\begin{array}{c} \mu_{j}\left(x_{p}\right)\cdot {\text{CF}}_{j}=\max\left\{\mu_{j}\left(x_{p}\right)\cdot {\text{CF}}_{j}R_{j}\right\}. \end{array}$$

Thus, the winner rule has the maximum product of the compatibility and the certainty factor CF_*j*_.

Each fuzzy rule is encoded into a specific string. Fifteen symbols are used for representing the 14 antecedent fuzzy sets in Figure 1 plus* don't care.* These symbols are as follows:

0= do not care (DC), 1 = small (S^2^), 2 = large (L^2^), 3 = small (S^3^), 4 = medium (M^3^), 5 = large (L^3^), 6 = small (S^4^), 7 = medium small (MS^4^), 8 = medium large (ML^4^), 9 = large (L^4^), a = small (S^5^), b = medium small (MS^5^), c = medium (M^5^), d = medium large (ML^5^), and e = large (L^5^).

For example, fuzzy rule “0150b0⟶0” denotes that the fuzzy rule “If *x*_2_  is S^2^ and *x*_3_is L^3^and *x*_5_ is MS^5^, then class is 0.”

### 2.3. Particle Swarm Optimization

#### 2.3.1. RAM Concept

At first, the RAM concept, which has been used in PSO algorithm, must be explained. RAM term in this work is the same as what is defined in [19] by Saniee et al. Here, we describe it briefly.

If *R*_*A*_ is a fuzzy rule with *n* antecedents, then *R*_*A*_ is as follows:

(5) $$\begin{array}{c} R_{A}=\left(a_{i}\right),\;i=1,2,\ldots ,n. \end{array}$$

Rule antecedent modification (RAM) operator or RAM(*k*, *A*_*j*_) is defined as follows.

RAM operator stands for substitution of the existing linguistic value of the rule *k*th precondition with the *j*th linguistic value. Thus, by applying RAM operator, a new fuzzy rule is made according to the following equation:

(6) $$\begin{array}{c} R_{A}^{\prime }=R_{A}+\text{RAM}\left(k,A_{j}\right). \end{array}$$

So, the symbol “+” in (6) has a new concept. For example, if we have a classification problem with five inputs, a fuzzy rule for this problem is like the following equation:

(7) $$\begin{array}{c} R_{A}=\left({\text{S}}^{5},{\text{ML}}^{4},{\text{L}}^{2},{\text{M}}^{3},\text{DC}\right). \end{array}$$

Suppose that the RAM operator is RAM(3, *A*_1_). The result of applying this operator is shown in the following equation:

(8) $$\begin{array}{c} R_{A}^{\prime }=R_{A}+\text{RAM}\left(3,A_{1}\right)=\left({\text{S}}^{5},{\text{ML}}^{4},{\text{S}}^{2},{\text{M}}^{3},\text{DC}\right). \end{array}$$

Rule antecedent modification sequence (RAMS) is built with more than one RAM operators which are applied, respectively, as follows:

(9) $$\begin{array}{c} \text{RAMS}=\left({\text{RAM}}_{1},{\text{RAM}}_{2},\ldots ,{\text{RAM}}_{n}\right). \end{array}$$

Note that the order of RAM operators in RAMS is very important.

When it is said that one RAMS acts on a solution, it means that all the RAM operators of it act on the solution in order.

Operator ⊕ is defined for merging two RAM sequences into a new RAM sequence.

Consider that RAMS1 and RAMS2 are two RAM sequences that operate on *R*_*A*_, respectively. With this act, a new rule *R*_*A*_′ is obtained. Now, suppose that there is another RAMS sequence RAMS′ operating on solution *R*_*A*_ with the same result, *R*_*A*_′. Under these circumstances, RAMS′ is described as follows:

(10) $$\begin{array}{c} {\text{RAMS}}^{\prime }=\text{RAMS}1⊕\text{RAMS}2. \end{array}$$

RAMS1 ⊕ RAMS2 and RAMS′ are in the same equivalent set.

In an equivalent set, the RAMS having the lowest number of RAM operators is called basic RAM sequence (BRAMS).

Consider that *R*_*N*_ and *R*_*M*_ are two solutions. Next definition is about BRAMS_*M*→*N*_; that is,

(11) $$\begin{array}{c} {\text{BRAMS}}_{M\to N}=R_{N}-R_{M}. \end{array}$$

BRAMS_*M*→*N*_ in previous equation is a basic RAM sequence which acts on *R*_*M*_ to obtain the solution *R*_*N*_.

#### 2.3.2. PSO Algorithm

Particle swarm optimization (PSO) is an evolutionary computation method for optimization that was developed by Kennedy and Eberhart in 1995 [20]. It is inspired by social behaviour of bird flocking or fish schooling and swarm theory.

This algorithm works by simultaneously maintaining a number of candidate solutions in the search space. Each candidate solution is considered as a particle “flying” in the *n*-dimensional search space to find the best solution. In all of the iterations of algorithm, each candidate solution, or, in other words, each particle, is appraised by the objective function, and the fitness of that solution is calculated. At first, like the GA, the PSO algorithm is initialized with a population of random solutions in the search space. PSO also needs only the information about the fitness values of the particles in the population. This algorithm simply calculates the fitness values of the individuals by applying the objective function. In comparison with genetic algorithm, individuals in PSO have memory so that the information of the particles with better solutions is maintained by all individuals. In other words, it creates a constructive cooperation between the individuals, and the individuals share information between themselves.

All the individuals have a location vector *x*_*i*_ and a velocity vector *V*_*i*_. Location of each individual is updated at each iteration considering its personal best position (*P*_*i*_) and the best position among all individuals (*P*_*g*_) at each iteration.

The PSO algorithm only consists of three steps which are repeated until some stopping condition is met [21].

(1) Calculate the fitness value of each particle.
(2) Update local and global best positions and fitness.
(3) Calculate the new velocity and position of each particle with regard to
  (12) Vi(t+1)=ωVi(t)+c1×(Pi(t)−xi(t))+c2×(Pg(t)−xi(t)),xi(t+1)=xi(t)+Vi(t+1),

where *ω* is the inertia weight that is applied to control the influence of the previous history of the velocities on the new velocity.

*c*_1_ and *c*_2_ are acceleration coefficients; *r*_1_ ~ *U*(0,1) and *r*_2_ ~ *U*(0,1) are random constants in the range [0,1] and are uniformly distributed. This velocity-updating method allows the particles to search around their individual best position *P*_*i*_ and the global best position *P*_*g*_.

### 2.4. Ensemble Based Methods

Machine learning is a set of nonparametric statistical methods. By simulating human idea process, it gives a smart structure. In comparison to other statistical models, in the field of machine learning, a model is made without any assumption. The most important concept in this field is the generalization ability. It confirms how well the result learned by a training data set can be applied to the unsighted test data set. Ensemble learning is a class model in the field of machine learning. Due to its stable and accurate performance, this method is popular for prediction and classification. In contrast with ordinary methods, in machine learning, which tries to construct only one learner from the training data, ensemble methods try to construct several learners and to combine them. In general, the generalization ability of an ensemble learner is better than a single learner.

In fact, ensemble techniques are more popular because of their ability to strengthen weak learners [22].

Bagging, Adaboost, Logitboost, Random Forest, and so forth are various ensemble techniques, whereas Random Forest and bagging act in parallel and the rest act in sequential act in parallel are boosting acts in sequential.

An ensemble technique has two steps: (1) generating individual members and (2) right combining individual members' outputs to find a new output [12].

## 3. The Proposed Methodology and Implementation

### 3.1. Boosting Algorithm

Boosting algorithm was offered by Freund and Schapire [23, 24]. It is a method of producing very accurate prediction rules by combining several “weak” learners that can only be moderately accurate. Boosting algorithm obviously modifies the distribution of the training data that has been given to each single classifier by the weights of training samples. At first, all training samples are given the same weight. During the boosting process, those weights are changed based on the error that current classifier has on the training data. The weights of those samples that are correctly classified are reduced, while the weights of those that are misclassified or not covered by the classifier remain unchanged. The weight *w*_*k*_ indicates the importance of the *k*th instance in current training data set. When a weak classifier is run, the weights *w*_*k*_ of the correctly classified instances are decreased; thus, the next weak classifier pays more attention to the misclassified or not covered instances due to their higher weights.

The initial weights of all examples are the same and are equal to value 1. The PSO algorithm is repeatedly called on the current training data by the boosting algorithm. The error ER_*R*_*T*__ of the fuzzy rule *R*_*T*_, produced by PSO algorithm in *T*th run, is calculated by the boosting algorithm. The parameter ER_*R*_*T*__ is evaluated by measurement matching *μ*(*x*_*k*_) which determines the measure of matching between the *K*th training instance and the fuzzy rule R_*T*_ antecedent as well as its weight *w*_*k*_. Consider

(13) $$\begin{array}{c} {\text{ER}}_{R_{T}}=\frac{\sum_{k\;|\;c_{k}\neq C_{i}}^{}w_{k}\mu \left(x_{k}\right)}{\sum_{k}^{}w_{k}\mu \left(x_{k}\right)}. \end{array}$$

The goal of this intelligent system is to find classification rules that classify accurately the current distribution of the training data.

In normal form of Adaboost algorithm, only for the correctly classified instances, the weights are reduced. The weight of a correctly classified instance is decreased by factor *α* ^*k*^. Consider

(14) $$\begin{array}{c} w_{k}\left(t+1\right)=\{\begin{array}{cc} w_{k}\left(t\right), & C_{i}\neq C_{k}\\ w_{k}\left(t\right)\times \alpha^{k}, & C_{i}=C_{k}, \end{array} \end{array}$$

(15) $$\begin{array}{c} \alpha^{k}={\left(\frac{1}{1+\text{EXP}(\mu (x_{k})/\left({\text{ER}}_{R_{T}}+w_{k}\right))}\right)}^{\mu (x_{k})}. \end{array}$$

The factor *α* ^*k*^ is obtained based on three parameters *μ*(*x*_*k*_), ER_*R*_*T*__, and the weight *w*_*k*_. According to (14) and (15), as the instance and the rule antecedent are more matched, the weight of the instance is more reduced. Also, the weights of the instances, which are correctly classified by a rule with less error or are better known in previous iteration, are more reduced. The weights of the misclassified or uncovered instances have been left unchanged.

### 3.2. Fuzzy Rule Extraction with PSO Algorithm

PSO algorithm has been used in various data mining problems such as clustering and classification [25–27]. In this paper, PSO algorithm extracts appropriate rules for a classification problem.

Population *P* with *L* particles is defined as follows [28]:

(16) $$\begin{array}{c} P=\left[\begin{array}{c} p_{1}\\ p_{2}\\ \vdots \\ p_{h}\\ \vdots \\ p_{L} \end{array}\right]=\left[\begin{array}{cc} r_{1} & g_{1}\\ r_{2} & g_{2}\\ \vdots & \vdots \\ r_{h} & g_{h}\\ \vdots & \vdots \\ r_{L} & g_{l} \end{array}\right], \end{array}$$

where $p_{h}=\left[\begin{array}{cc} r_{h} & g_{h} \end{array}\right]$ is a particle of population that defines a set of fuzzy rules. $r_{h}=\left[\begin{array}{ccccccc} r_{1}^{h} & r_{2}^{h} & r_{3}^{h} & \cdots & r_{j}^{h} & \cdots & r_{B}^{h} \end{array}\right]$ includes a set of candidate fuzzy rules. It defines the position of a particle where *B* is a positive integer variable to set the maximum number of fuzzy rules that can be produced. The velocity vector defines as $V_{h}=\left[\begin{array}{ccccccc} v_{1}^{h} & v_{2}^{h} & v_{3}^{h} & \cdots & v_{j}^{h} & \cdots & v_{B}^{h} \end{array}\right]$ that *v*_*j*_^*h*^ is a BRAMS. The parameter vector $g_{h}=\left[\begin{array}{cccccc} g_{1}^{h} & g_{2}^{h} & \cdots & g_{j}^{h} & \cdots & g_{B}^{h} \end{array}\right]$ is used in order to reduce the number of fuzzy rules. If the parameter *g*_*j*_^*h*^ is less than or equal to a user-defined maximum threshold, the rule *r*_*j*_^*h*^ remains in fuzzy rule set; otherwise, it is eliminated. In initialization step, this parameter is random in [0, 1]; then, it is calculated according to (17) in each iteration. Consider

(17) $$\begin{array}{c} g_{j}^{h}=\frac{{\text{NMP}}_{r_{j}^{h}}\left(w\right)}{{\text{NCP}}_{r_{j}^{h}}\left(w\right)}, \end{array}$$

(18) $$\begin{array}{c} {\text{NMP}}_{r_{j}^{h}}\left(w\right)=\underset{k\;|\;c_{k}\neq C_{j}}{\sum }w_{k}, \end{array}$$

(19) $$\begin{array}{c} {\text{NCP}}_{r_{j}^{h}}\left(w\right)=\underset{k\;|\;c_{k}=C_{j}}{\sum }w_{k}. \end{array}$$

In fact, NMP_*r*_*j*_^*h*^_(*w*) is the total weight of the instances that have been incorrectly diagnosed and NCP_*r*_*j*_^*h*^_(*w*)  is the total weight of the instances that have been correctly diagnosed by the rule *r*_*j*_^*h*^.

Suppose that *r*_*h*_ is the number of acceptable fuzzy rules; then, index of these rules is *I*_*r*_ ∈ {1, 2 … *B*}, *r* = 1, 2,…, *r*_*h*_, and the acceptable fuzzy rule set is as {*r*_*I*_1__^*h*^, *r*_*I*_2__^*h*^,…, *r*_*I*_*r*__^*h*^,…, *r*_*I*_*r*_*h*___^*h*^}.

The position of a particle in iteration *T* is considered as  *r*_*h*_(*T*); in this case, its next position will be as follows:

(20) rh(T+1)=rh(T)+Vh(T)=[rI1h+vI1h,rI2h+vI2h⋯rIrh+vIrh⋯rIrhh+vIrhh].

The next velocity of each particle is calculated by the following equation:

(21) Vh(T+1)=ω×Vh(T)⊕α×(Lh(T)−rh(T))  ⊕β×(Lg(T)−rh(T)).

*L*_*h*_(*T*) is the best ever existing position experienced by the particle and is called local best position. Also, *L*_*g*_(*T*) is called global best position and is the best position ever experienced by all the particles.

### 3.3. The En-PSO2 Approach

In a number of researches, combinations of nature-inspired algorithms and ensemble methods have been used for rule extraction. For example, Ant-Miner method can be expressed. In Ant-Miner, a series covering method is followed to find a set of classification rules covering all, or approximately all, the training instances [29]. Another example is the study that is referred to in [30] in which a boosting algorithm is combined with genetic algorithm to extract classification rules.

In this section, we will describe the details of En-PSO2 approach for generating the classification fuzzy rules for detecting coronary artery disease.

**Figure 3:**
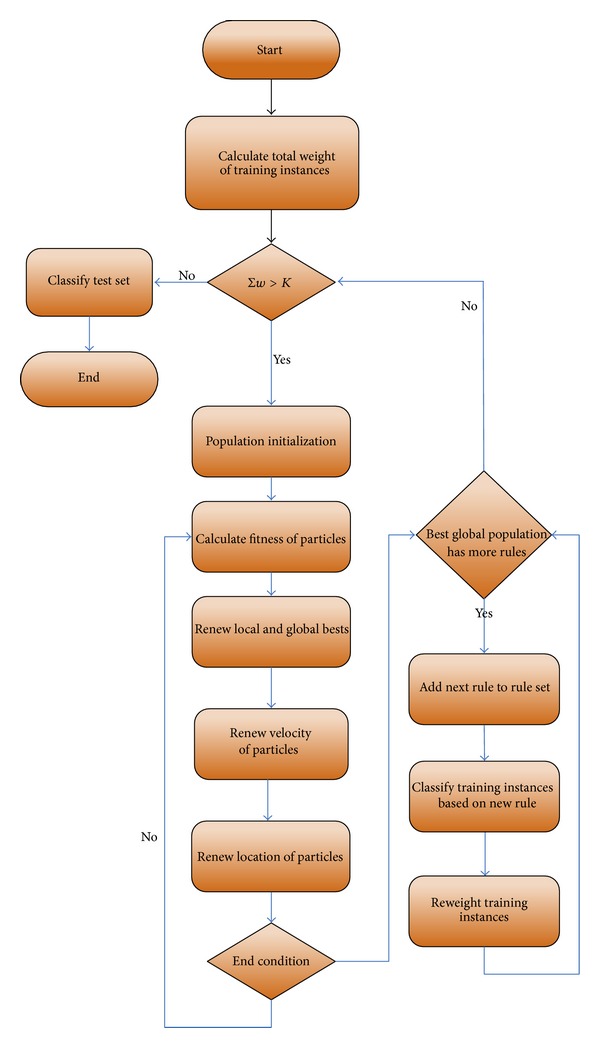
Total flowchart of the expert system of coronary artery detection. *K* is the allowable amount for total weights of training examples.

In this method, the PSO algorithm that has been described in Sections 2 and 3.2 runs several times. In previous works, in En-PSO algorithm, each time that PSO algorithm was run, only one fuzzy rule was added to the rule set, but, in En-PSO2, each time that PSO is run, several fuzzy classification rules are extracted and added to the fuzzy rule set. Maximum number of the fuzzy rules that may be extracted in each run is equal to parameter B (defined in previous section). The new boosting method, described in Section 3.1, considers the collaboration between the fuzzy classification rules that are extracted from PSO algorithm. After a rule is added to rule set, the weights of those instances that have been correctly classified with a recently extracted rule are reduced by boosting algorithm and, for the remaining instances, the weight will not be changed to increase their chances in next runs. Thus, the En-PSO2 is biased to find those fuzzy classification rules that complete the existing fuzzy rule set and rectify its shortages. The pseudocode of En-PSO approach is shown in Pseudocode 1. In this pseudocode,   *K* is the allowable amount for total weights of training examples.  Figure 3 presents a flowchart showing how En-PSO2 algorithm works.

## 4. Experimental Result

In this research, we had two classes: 1 and 0; 1 refers to the healthy people and 0 is for patients who are subject to possible CAD. To test this method, we used UCI coronary artery data set which was described in Section 2.

This data set has 920 instances: 509 instances with coronary artery disease and 411 instances without coronary artery disease.

We have run En-PSO2 method with various parameters and at last we set them as in Table 2.

To achieve more reliable results, in partitioning training and test sets, *K*-fold cross-validation technique was used. In the mentioned method, the main data set is randomly divided into *K* partitions. Here, the classification method is run ten times. Each time, the next subset is considered as the validation and the remaining *k* − 1 partitions are used as training data. In fact, in *K*-fold cross-validation, each of the *K* partitions is used once as validation data. The *K* results are averaged to a single estimation. Different tests on various data sets have shown that 10 is almost the best number of folds to get the best approximation of error [31].

Different evaluation criteria have been used in data mining and machine learning to test methods performances. The classification accuracy is the most common evaluation criterion used in data mining field. We have considered five criteria to evaluate the performance of the proposed method: accuracy, specificity, sensitivity, precision, and *F*-measure.

To calculate these measures, a well-known matrix called confusion matrix (contingency table) is formed. This matrix represents the classification results.

When the confusion matrix was constructed, the accuracy, specificity, sensitivity, precision, and *F*-measure can be easily calculated as the following [15]:

(22) accuracy=(TP  +  TN)(TP  +  FP  +  TN  +FN),specificity=TN(TN  +  FP),sensitivity=recall=TP(TP  +  FN),precision=TP(TP+FP),F-measure=2∗precision∗recall(precision+recall).

*True Positive (TP)*. It is the number of correct predictions with CAD that is diagnosed as patient by the angiography. 

*True Negative (TN)*. It is the number of correct predictions as normal with CAD that is labelled as healthy by the angiography.

*False Negative (FN)*. It is the number of incorrect predictions as normal with CAD that is diagnosed as patient by the angiography. 

*False Positive (FP)*. It is the number of incorrect predictions as a patient that is labelled as a healthy person by the angiography.

Classification accuracy is calculated by the ratio of the number of the instances correctly classified to the total number of samples.

Precision measures the percentage of the actual patients (i.e., true positive) between the patients that got declared CAD; recall measures the percentage of the actual patients that were discovered; *F*-measure balances between precision and recall. Specificity measures the percentage of patients without disease that can be correctly eliminated.

Table 3 shows the confusion matrix obtained by the proposed system. We achieved accuracy of 85.76 on UCI data set of coronary artery disease. All the five performance measures, accuracy, sensitivity, specificity, precision, and F-measure, for En-PSO2 have been shown in Table 4. This method has generated in average 21.2 rules. The comparison between rule-based methods is presented in Table 5. As can be seen among all the rule-based methods for detection of CAD, the minimum number of rules is allocated to En-PSO2. Also, the accuracy of this method is in second place after En-PSO. In addition, the average length of rules is 3.01. These results suggest that the interpretability of the proposed method is very high since the comprehensibility of a rule-based expert system with a few if-then rules is much more than a system with large number of rules.

**Figure 4:**
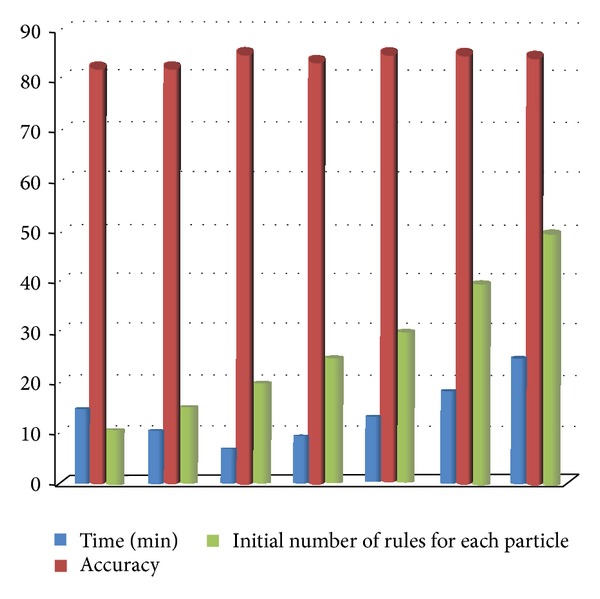
Time and accuracy changes based on increasing amount of *B*.

In Figure 4, two factors, time and accuracy of the method, are compared with the increasing amount of initial number of rules for each particle (*B*). As can be seen maximum accuracy is achieved by setting 30 for *B*. This negligible increase in accuracy (approximately 0.03) is obtained with a time difference of about 6.5 minutes. Therefore, we have set 20 for *B*.

**Figure 5:**
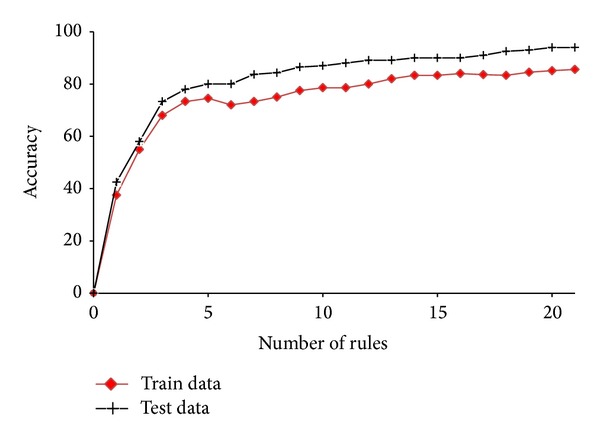
Average accuracy obtained on train and test data sets from adding each rule to the rule set.

Figure 5 illustrates the average accuracy after adding each rule to rule set. Given that the method which we have chosen to test the proposed method is 10-fold cross-validation, we do not have a single rule set, and we have 10 rule sets. Therefore, in Figure 5, we have calculated the average of accuracy. As can be seen, the average accuracy on the train data set is always ascending, but the slope of graph is declining. In the earlier runs, the extracted rules cover the samples easier and reduce their chances for selection in the next runs. So, with increasing the number of runs, the rules are more specific and cover the less number of samples; thus, increasing accuracy is less.

Table 6 compares the classification accuracy, sensitivity, specificity, and number of rules between proposed method and previous methods. This comparison shows that En-PSO2, both in terms of performance and the interpretability, is better.

It is important to note that the computational complexity of our method is *O*(*P*∗*R*∗*F*); *P* denotes number of particles; *R* addresses the number of patients records; and *F* is number of features. In comparison to other works, it seems that our algorithm is more complex and needs more time to extract its knowledge base (fuzzy if-then rule). However, since the testing time is very fast (*O*(*F*)) and the extracted rule set is very accurate and interpretable, we can accept the final performance of this complex learning algorithm.

## 5. Conclusion

In a number of researches, diagnosis of CAD has been considered. These researches have applied different techniques to the given problem and attained high classification accuracies on the UCI data sets. In a number of these researches, only determination of patient or normal has been considered while, in some other, the extraction of appropriate rules is also considered.

In this research, a fuzzy boosting PSO approach has been proposed to generate appropriate rules for detection of CAD. The proposed boosting algorithm helps to produce optimal rules to cover more instances.

In our previous works [18, 32], in each run, one rule was extracted and added to rule set. In this work, each individual in PSO algorithm includes a set of rules. Therefore, each time the PSO algorithm is run, several rules are added to rule set. This has two results. (1) Algorithm will be completed faster if appropriate values for the parameters are set. This is because each time PSO algorithm is run, several rules can be added to rule set and this makes the process of the instance covering faster. (2) There is more coordination between production rules. In previous method, the extracted rule in each run may be optimal in terms of covering instances but may not be coordinate between existing rules in rule set. Consequently, the accuracy of the method may be reduced. In En-PSO2, in each run, several rules are extracted which are compatible. So, in this method, rules are more coordinate.

This method, in addition to being competitive with other approaches in terms of accuracy, produces an average of 21.2 rules that is the minimum number of rules for the detection of the mentioned disease. Moreover, the average length of rules is 3.01. These results show that the interpretability of the proposed method is very high (in comparison to [18, 33, 34], whose methods produce more number of if-then rules to detect CAD).

## Figures and Tables

**Pseudocode 1:**
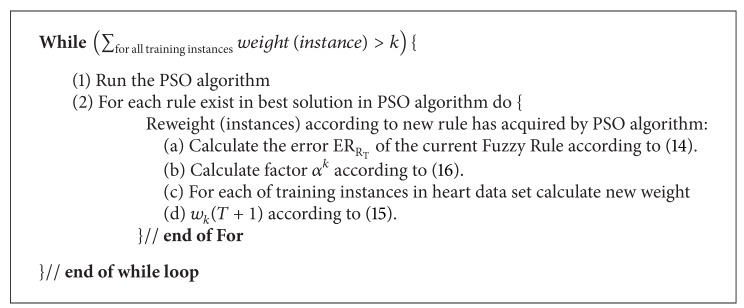
The pseudocode of En-PSO2 approach.

**Table 1:** Summary of attributes (UCI heart disease data base).

Attribute	Description	Value description
age	Age	Numerical
sex	Sex	1: if male; 0: if female
cp	Chest pain type	1: typical angina
		2: atypical angina
		3: nonanginal pain
		4: asymptomatic
trestbps	Resting systolic blood pressure on admission to the hospital (mmHg)	Numerical
chol	Serum cholesterol (mg/dL)	Numerical
fbs	Fasting blood sugar over 120 mg/dL?	1: if yes
		0: if no
restecg	Resting electrocardiographic results	0; normal
		1: having ST-T wave abnormality
		2: having LV hypertrophy
thalach	Maximum heart rate achieved	Numerical
exang	Exercise induced angina?	1: if yes
		0: if no
oldpeak	ST depression induced by exercise relative to rest	Numerical
slope	The slope of the peak exercise ST segment	1: upsloping
		2: flat
		3: downsloping
ca	Number of major vessels colored by fluoroscopy	Numerical
thal	Exercise thallium scintigraphic defects	3: normal
		6: fixed defect
		7: reversible defect
num	Diagnosis of heart disease (angiographic disease status/presence of coronary artery disease (CAD))	0: if less than 50% diameter narrowing in any major vessel (CAD, no)
		1: if more than 50% (CAD, yes)

**Table 2:** Parameter setting for the proposed En-PSO2.

Parameter	Value
Swarm size	25
Number of iteration	Until the global best does not change after 5 iterations
*ω*	1/iteration number
c_1_	Random (between 0 and 1)
*c*_2_	Random (between 0 and 1)
*K*	12
*B*	20

**Table 3:** The obtained confusion matrix.

Actual result			
Patient	Healthy		
90	419	Healthy	Classifier result
370	41	Patient	

**Table 4:** Performance measures of En-PSO2 according to confusion matrix.

Measure name	Accuracy	Sensitivity	Specificity	*F*-measure	Precision
Amount (%)	85.76	90.02	82.31	86.48	91.08

**Table 5:** Comparison between rule-based methods.

Rule-based methods	Accuracy	Number of rules
Decision tree [33]	85.6	83
Support based [33]	84.4	27
Pearson [34]	84.5	27
RST [33]	85.2	27
En-PSO [18]	85.97	25.3
**En-PSO2 (this study)**	**85.76**	**21.2**

**Table 6:** Comparison between classification algorithms according to accuracy, sensitivity, specificity, and number of rules.

Method	Accuracy	Sensitivity	Specificity	Number of rules
Decision tree [35]	78.91	72.01	84.48	—
LTF-C [36]	81.2	—	—	—
Bagging [35]	81.41	74.93	86.64	—
*k*-NN [33]	81.5	—	—	—
Bayesian model [37]	82	87	—	—
Decision tree (C4.5) [37]	82.5	87.17	—	—
SVM [37]	82.5	88	—	—
RST [33]	85.2	—	—	27
ANN [38]	85.53	—	—	—
Decision tree [33]	85.6	—	—	83
NN-Alizadeh [15]	85.43	90.2	73.5	—
En-PSO2 (this study)	85.76	90.02	82.31	21.2
